# Communication in necrophagous Diptera larvae: interspecific effect of cues left behind by maggots and implications in their aggregation

**DOI:** 10.1038/s41598-018-21316-x

**Published:** 2018-02-12

**Authors:** Quentin Fouche, Valery Hedouin, Damien Charabidze

**Affiliations:** CHU Lille, EA 7367 - UTML - Unite de Taphonomie Medico-Legale, University of Lille, 59000 Lille, France

## Abstract

Necrophagous Calliphoridae breed in vertebrate carrion. Their larvae aggregate and form large masses of individuals. These aggregated larvae can reach adulthood faster than scattered larvae, increasing their chances of survival. Furthermore, the gathering of larvae of different species suggests possible interspecific aggregation vectors. In this context, the effect of larval ground-left cues on larvae of *Calliphora vomitoria* and *Lucilia sericata* was studied. We used video tracking to follow larvae placed in binary choice tests. We observed (1) a preference of both species for a side marked by conspecific or heterospecific larvae compared to an unmarked side, (2) a preference of *L. sericata* larvae for a conspecific-marked side compared to a heterospecific-marked side but only at high concentration of cues and (3) a preference of both species for the side marked by the greater number of larvae. These results demonstrate that larvae leave a mark locally which is retentive, has an interspecific range, has an effect proportional to its intensity and whose strength varies depending on the emitting species. According to the self-organization theory, this mark could enhance larval gathering and promote interspecific aggregations. While not yet demonstrated, an interspecific Allee effect could explain the interspecific association of necrophagous calliphorid larvae.

## Introduction

Many living organisms form aggregates. These groups of high density exist in various taxa but are especially common and well-known in arthropods such as woodlice^1^ or social insects^2,3^. The benefits of such a group formation (i.e., aggregation) include reduced risk of predation^4,5^, protection against environmental conditions^6^ and better food assimilation^7^. Under natural conditions, aggregates are mostly composed of individuals of the same species (i.e., intraspecific aggregates) but can also gather two or more different species (i.e., interspecific aggregates)^3^.

Aggregation can result from two main processes. Non-social aggregation refers to the gathering of individuals under the influence of environmental heterogeneity^8^. On the other hand, social aggregation occurs as a result of attraction between individuals. This process requires aggregation vectors, i.e., visual, auditory, tactile or chemical stimuli efficient at a variable range^8,9^. In most cases, social aggregation includes a self-organizing process, defined as the emergence of complex collective behavior from simple and repeated interactions between individuals^2,10^. During aggregation, local individual behavior acts as a positive feedback for conspecifics and this feedback amplifies the aggregative behavior, ultimately leading to the emergence of the collective decision^2,10–12^. While intraspecific aggregation has already been the subject of numerous studies^13^, the formation of interspecific aggregates and corresponding aggregation vectors are still poorly understood^3^.

Among Diptera, necrophagous Calliphoridae larvae grow and feed on vertebrate carrion^7,14^. This rich and abundant resource allows fast and efficient larval development. During the feeding instars, larvae aggregate and form huge masses that can contain hundreds to thousands of individuals^7^. Furthermore, several species in this family are known to aggregate together and form mixed-species groups^3,15–18^. A striking consequence of larval aggregation, the so-called *maggot-mass effect*, is a local temperature increase which can reach 20 °C above ambient. This heat production is proportional to the number of larvae in the aggregate^18,19^. As the developmental speed of larvae increases with temperature^20^, aggregated larvae benefiting from the larval-mass effect can reach adulthood faster than isolated individuals^21–24^. This reduced development time likely increases the chances of survival of larvae, while aggregation confers other benefits such as better nutrients absorption and protection against predators and parasites^5,7^. Deleterious effects linked to thermal stress, overcrowding and competition between individuals have also been reported^7,24,25^.

A recent study demonstrated larval social aggregation in two blowfly species, the common green bottle fly *Lucilia sericata* and the blue bottle fly *Calliphora vomitoria*^12^. This result suggests possible aggregation vectors shared between the two species^12^. The authors also demonstrated that *L. sericata* larvae leave on the ground a cuticular mark having a retentive effect on congeners^26^. According to the authors, this could promote aggregation of larvae and thus constitute an aggregation vector^26^.

The present study investigates the interspecific effect of the cuticular ground-left cues of *C. vomitoria* and *L. sericata* larvae. Three hypotheses were experimentally tested using *in vitro* binary choice tests: (1) the cues locally left by larvae affect the behavior of larvae of the other species; (2) these heterospecific cues have an effect similar to that of homospecific cues; and (3) the effect of the cues increases in proportion to their concentration.

## Material and Methods

### Biological material

Larvae were obtained from adult flies collected in the field and reared in the laboratory. Adults of *C. vomitoria* and *L. sericata* were reared separately in a 50 × 50 × 50 cm insectarium kept at room temperature (20 ± 2 °C) under a natural light cycle. Water and sugar were provided *ad libitum*. Twenty grams of fresh chopped beef liver were introduced each day to provide the protein required for vitellogenesis and to trigger egg laying. Eggs were collected daily and deposited in a plastic box (108 × 83 × 64 mm) containing 100 g of chopped beef liver. This box was placed in an incubator (Pol-Eko-Aparatura model ST BASIC) at a temperature of 20 ± 1 °C. Only young third instars (8 ± 1 mm) were used for experiments; this meant five-day old larvae for *L. sericata*^27^ and seven-day old larvae for *C. vomitoria*^28^.

### Binary choice test

**Figure 1:**
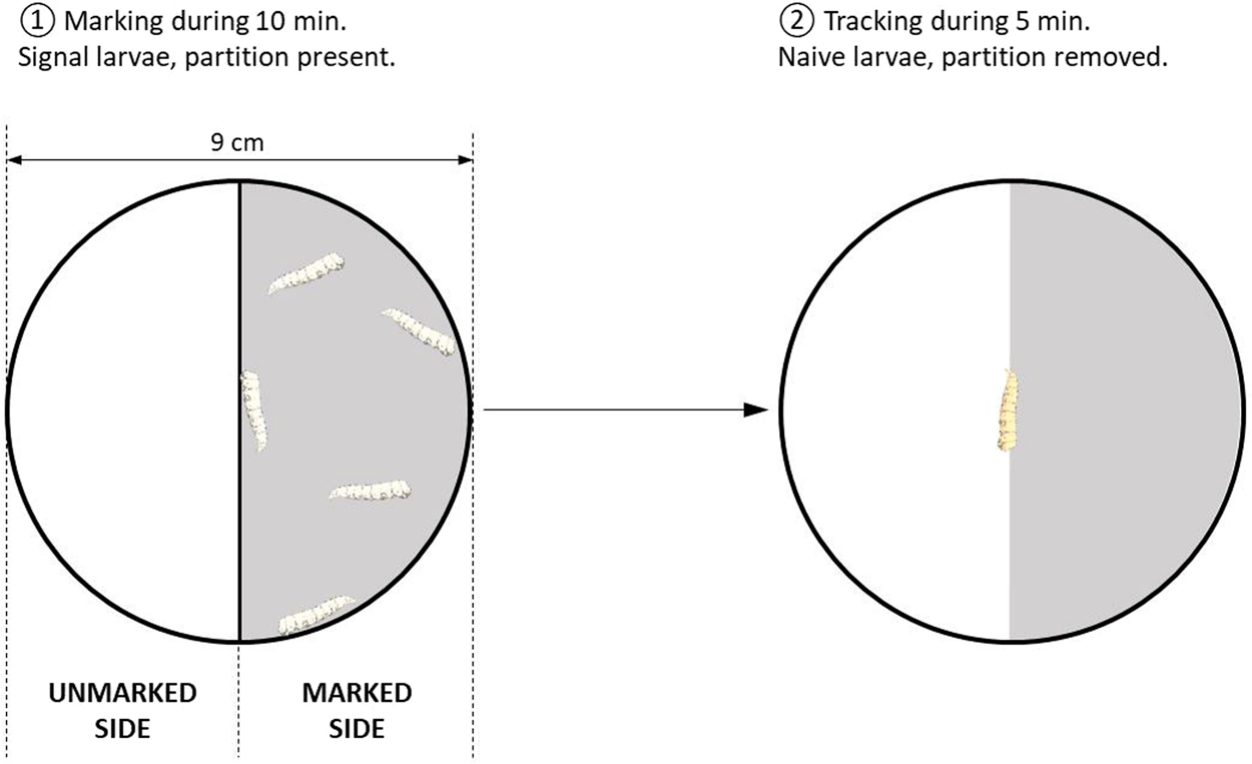
Binary-choice setup used during the first and second steps of trials. During the first step, the arena was divided in two. In this example, one side was marked by five larvae for 10 minutes (grey, marked side) while the other side remained blank (white, unmarked side). In the second step, the partition and the marking larvae were removed and a naive larva was placed into the center of the arena. Its displacements were then video-tracked for 5 minutes.

The effect of cuticular cues on larval behavior was studied using binary choice tests based on the method of Boulay *et al*. (2013)^26^. Larvae were placed in a Petri dish (2 cm in height, 9 cm in diameter) divided into two halves. The bottom of the arena was covered with moistened filter paper (Fig. 1). The dish was placed in an incubator (Liebherr, model FKS 1800) at 25 ± 2 °C and illuminated from below with a red light (630 nm) not visible to the larvae^29^. As the locomotor activity of the larvae is not linked to a circadian cycle^30^, experiments were performed daily between 13 h and 19 h. Controls showed that this experimental setup did not produce spatial bias (see Supplementary Fig. S1).

Each test was conducted in two steps: 1/marking the arena and 2/tracking the displacement of a “naive” (i.e., never tested) larva. In the first step (marking), the arena was divided into two halves using a plastic strip, thus creating 2 semicircles of 4.5 cm radius (Fig. 1). Five or 40 “marking” larvae were placed on one side for 10 minutes and allowed to crawl on the paper to leave their cues. The larvae and the plastic strip were then removed. In the second step (tracking), a naive larva was placed in the center of the arena (Fig. 1) and video-recorded for 5 minutes (Veditec camera, model VED-037, Resolution: 976 × 582). The orientation of the arena in the incubator was reversed between each test so that the marked side was positioned half of the time on the left and half of the time on the right. At the end of each trial, the arena was disassembled and thoroughly cleaned with 95% ethanol.

Before performing each test, larvae were kept at 25 ± 1 °C in a pillbox containing moistened pine sawdust for 30 minutes to remove food remains potentially present on their cuticle. An additional 3 h and 30 minutes confinement under the same conditions was applied to marking larvae in order to starve them and to avoid having them defecate on the filter paper during marking^26,31^. Complementary tests showed that this cleaning (4 h confinement with pine sawdust) was sufficient to remove any traces of food from the larval cuticle (see Supplementary Fig. S2).

Six different marking combinations were tested with 30 replicates performed for each. The conditions “control vs. 5 *L. sericata*” and “control vs. 5 *C. vomitoria*” were designed to test the ability of larvae to perceive and react to a conspecific or heterospecific cue. The combinations “5 *C. vomitoria* vs. 5 *L. sericata*” and “40 *C. vomitoria* vs. 40 *L. sericata*” were designed to test the ability of larvae to distinguish and respond differently to cues from different species. The combinations “5 *L. sericata* vs. 40 *L. sericata*” and “5 *C. vomitoria* vs. 40 *C. vomitoria*” were designed to test the effect of changing cue concentration.

### Data analysis

Video recordings were analyzed using Ethovision XT 8.5 software (Noldus Information Technology, Wageningen, The Netherlands). For each replication, the total duration, the total distance and the average speed in each side of the arena were calculated. The data between the two sides of the arena being paired, comparisons were performed using the Student’s t test for paired data when normality and homoscedasticity were present (respectively evaluated by the Shapiro’s test and the Fisher’s exact test), or using the Wilcoxon test when these conditions were not fulfilled. All analyses were performed with the R studio software (Version 0.98.1103), with a significance level set at α = 0.05. Two other parameters (the number of experiments in which the larva started to move in a side and the curvature of the larval path) were also calculated and compared between sides but, as the results were not significant (see Supplementary Figs S3 and S4), they were not shown in the present manuscript. Colormaps were generated using Ethovision to represent visually the differences of time spent by the larva between the different locations in the arena. Colors of the map represent the time spent at each coordinate of the arena with low wavelengths (e.g. red) indicating long retention time and high wavelengths (e.g. blue) indicating short retention time.

### Data availability

The datasets generated and analysed during the current study are available from the corresponding author on reasonable request.

## Results

### Larval detection of conspecific and heterospecific cues

**Figure 2:**
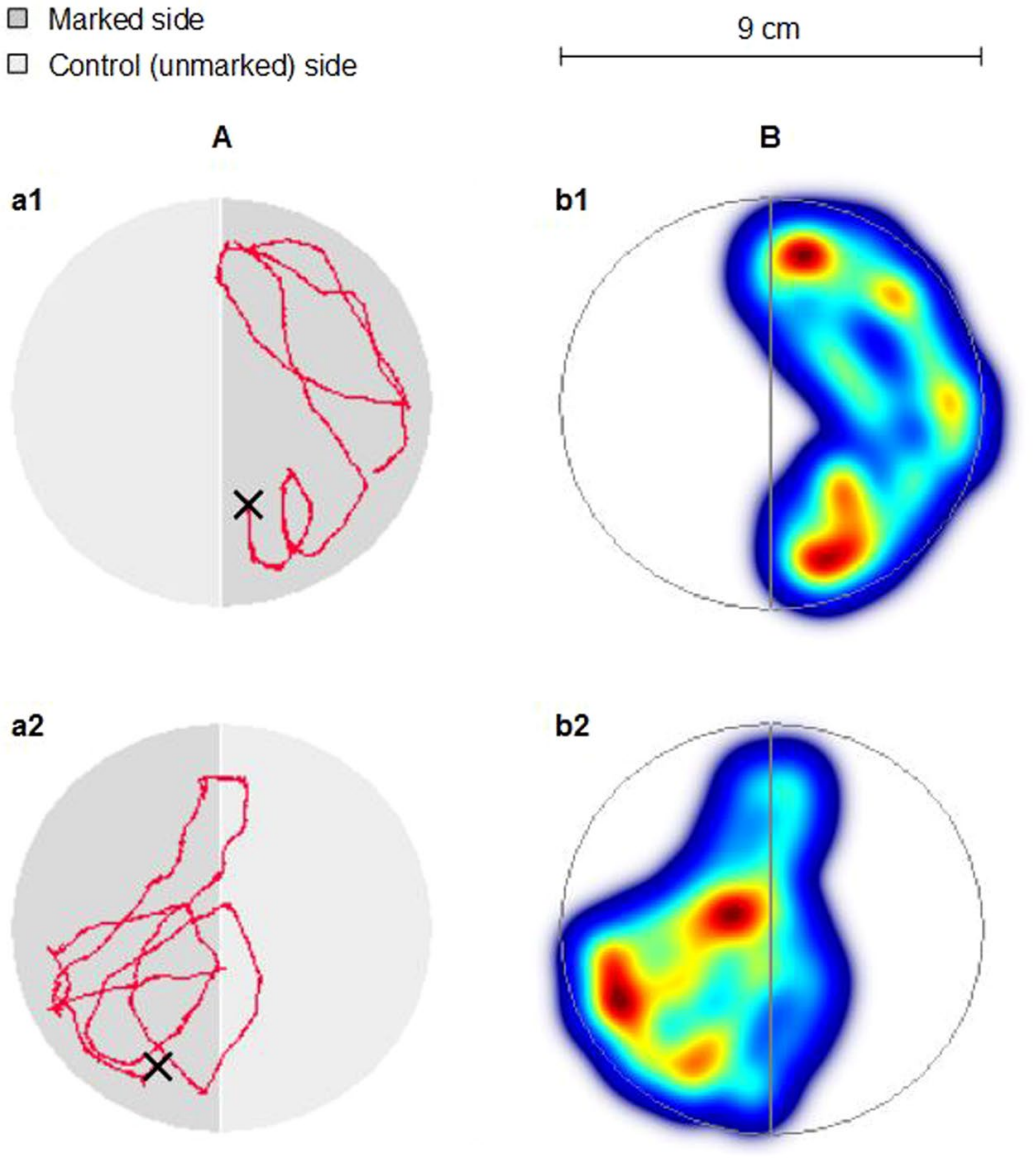
(**A**) Two examples of tracks (red, left side) performed by two different larvae (a1 and a2) having spent most of their time in the marked side (dark gray) than in the control side (unmarked side; light gray). Crosses indicate the place where the larvae was located when recording started (i.e. 2 or 3 seconds after being deposited at the center of the arena). (**B**) Colormaps related to the two tracks above (right side, b1 with a1, b2 with a2). The color gradient reveals the differences in the time spent by the larva at the different locations (from blue to red: from the least to the most of time spent).

**Figure 3:**
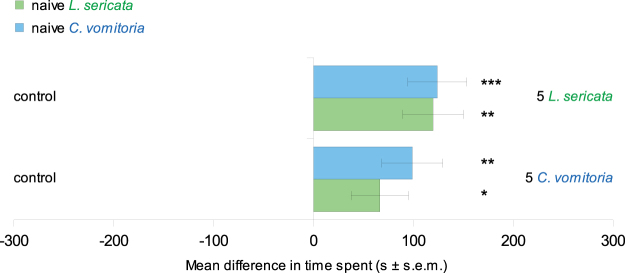
Mean differences (mean ± s.e.m.) in time spent between marked and non-marked sides. The time difference was calculated by subtracting the time spent on the non-marked side from the time spent on the marked side. The results obtained with naive *L. sericata* larvae are reported in green, while those for *C. vomitoria* are in blue. 30 replicates were performed for each condition. Student’s t test and Wilcoxon test, *P < 0.05, **P < 0.01, ***P < 0.001.

When only one side of the arena was previously occupied (i.e. marked) by five congeners, both *L. sericata* and *C. vomitoria* larvae spent significantly more time and travelled greater distances in the conspecific-marked side (Table 1, Figs 2 and 3). The same result was observed for the heterospecific cue: when one side of the arena was previously marked by five heterospecific larvae, both *L. sericata* and *C. vomitoria* larvae spent significantly more time and travelled greater distances in the heterospecific-marked side (Table 1, Fig. 3). In both conditions (conspecific and heterospecific marking), the average speed of the larvae did not differ significantly between marked and unmarked sides of the arena.

**Table 1:** Mean values of larval displacement in the two sides of the arena for heterogeneous marking conditions (different cues on the two sides of the arena).

Cues tested	side 1	control	control	5 *L. sericata*	40 *L. sericata*	5 *L. sericata*	5 *C. vomitoria*
	side 2	5 *L. sericata*	5 *C. vomitoria*	5 *C. vomitoria*	40 *C. vomitoria*	40 *L. sericata*	40 *C. vomitoria*
Time spent (s)	*L. sericata*	90 ± 15	117 ± 14	168 ± 14	194 ± 10	107 ± 11	108 ± 12
		210 ± 15	183 ± 14	132 ± 14	106 ± 10	193 ± 11	192 ± 12
		**V** = 73**	t = −2.36*	t = 1.27; NS	t = 4.57***	**V** = 69***	t = −3.51**
	*C. vomitoria*	88 ± 15	101 ± 15	141 ± 13	178 ± 17	93 ± 19	96 ± 11
		212 ± 15	199 ± 15	159 ± 13	122 ± 17	207 ± 19	204 ± 11
		V = 64***	V = 94**	t = −0.66; NS	t = 1.70; NS	V = 120*	t = −5.22***
Distance travelled (cm)	*L. sericata*	25 ± 4	36 ± 4	46 ± 4	54 ± 2	29 ± 3	31 ± 4
		56 ± 4	58 ± 5	38 ± 4	32 ± 3	52 ± 3	52 ± 3
		V = 73***	t = −2.68*	t = 1.18; NS	t = 4.44***	t = −3.81***	t = −3.29**
	*C. vomitoria*	30 ± 5	29 ± 5	46 ± 5	49 ± 5	26 ± 5	36 ± 5
		67 ± 5	61 ± 5	53 ± 5	32 ± 4	60 ± 6	70 ± 4
		V = 69***	V = 74***	t = −0.83; NS	t = 1.94; NS	V = 93**	t = −4.82***
Average speed (cm/s)	*L. sericata*	0.27 ± 0.01	0.32 ± 0.02	0.28 ± 0.01	0.29 ± 0.01	0.27 ± 0.01	0.28 ± 0.01
		0.27 ± 0.01	0.32 ± 0.01	0.29 ± 0.01	0.29 ± 0.01	0.27 ± 0.01	0.27 ± 0.01
		t = −0.32; NS	t = −0.73; NS	t = −0.85; NS	t = −1.38; NS	t = −0.01; NS	V = 252; NS
	*C. vomitoria*	0.32 ± 0.01	0.30 ± 0.02	0.34 ± 0.02	0.27 ± 0.01	0.31 ± 0.02	0.36 ± 0.02
		0.33 ± 0.01	0.31 ± 0.02	0.34 ± 0.01	0.28 ± 0.01	0.30 ± 0.02	0.36 ± 0.02
		t = −1.58; NS	V = 135; NS	V = 291; NS	t = −0.80; NS	t = 1.10; NS	t = 0.17; NS

For each condition, “side 1” is reported first and “side 2” is underneath; statistical values are reported on the last line. Asterisks (in bold) indicate a significant difference between the two sides (*P < 0.05; **P < 0.01; ***P < 0.001; NS: non-significant difference). 30 replicates were performed for each condition.

### Larval differentiation between cues

**Figure 4:**
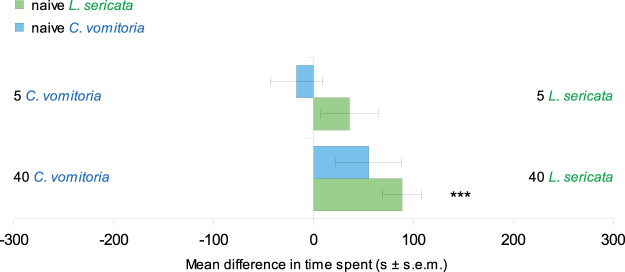
Mean differences (mean ± s.e.m.) in time spent between the sides marked by different species. The time difference was obtained by subtracting the time spent on the side marked by *C. vomitoria* larvae from the time spent on the side marked by *L. sericata* larvae. The results obtained with naive *L. sericata* larvae are reported in green, while those for *C. vomitoria* are in blue. 30 replicates were performed for each condition. Student’s t test and Wilcoxon test, ***P < 0.001.

Experiments comparing one side of the arena marked by five *L. sericata* larvae and the other side marked by five *C. vomitoria* larvae showed no difference in the time spent, the distance travelled or the average speed of the naive larvae between the two sides (Table 1, Fig. 4). This absence of choice was observed for larvae of the two tested species. However, when forty larvae were used for marking each side, *L. sericata* larvae spent significantly more time and travelled greater distances in the side marked by conspecifics (Table 1, Fig. 4). For *C. vomitoria*, the time spent and the distance travelled were also greater in the side marked by *L. sericata* larvae but these tendencies were not significant (time spent: Student t test, mean in the side marked by 40 *L. sericata* = 178 s, mean in the side marked by 40 *C. vomitoria* = 122 s, t = 1.70, P = 0.10; distance travelled: Student t test, mean in the side marked by 40 *L. sericata* = 49 cm, mean in the side marked by 40 *C. vomitoria* = 32 cm, t = 1.94, P = 0.06). For both species, the average speed did not differ significantly between the two sides of the arena.

### Effect of cue intensity

**Figure 5:**
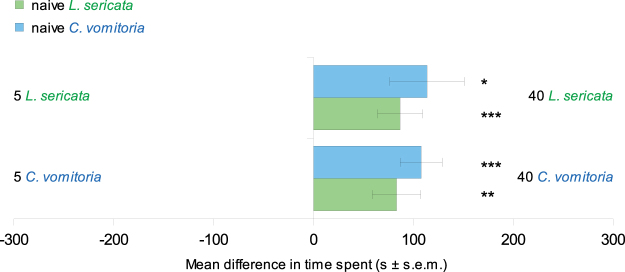
Mean differences (mean ± s.e.m.) in time spent in the side marked by 40 larvae minus the time spent in the side marked by 5 larvae. The results obtained with naive *L. sericata* larvae are reported in green, while those for *C. vomitoria* are in blue. 30 replicates were performed for each condition. Student’s t test and Wilcoxon test, *P < 0.05, **P < 0.01, ***P < 0.001.

Both *L. sericata* and *C. vomitoria* larvae spent significantly more time and travelled greater distance on the side marked by 40 larvae than on the side marked by 5 larvae. This was true for homospecific as well as heterospecific tests (Table 1, Fig. 5). In both cases, the average speed of the larvae did not differ significantly between the two sides of the arena.

## Discussion

This study demonstrates (1) a preference of *L. sericata* and *C. vomitoria* larvae for the side marked by larvae (conspecific or heterospecific), (2) a preference of *L. sericata* larvae for the side marked by conspecifics compared to the side marked by the other species and (3) a preference of both species for the side marked by a greater number of larvae (conspecific or heterospecific).

During tests comparing a larval-marked side to a non-marked side, the larvae consistently favored the marked side. This choice was observed for both conspecific and heterospecific marking. This result demonstrates that larvae can perceive the former presence of other larvae of both species. This detection induced a longer stay and greater distance travelled in the marked side, without change in the average speed. Accordingly, the cues left by larvae appear to have a retentive effect on other larvae. An attractive effect could also occur, but this cannot be evidenced by the present results. These results agree with the retentive effect of conspecific cues which have already been demonstrated in *L. sericata* by Boulay *et al*. (2013)^26^ and, confirming the two first hypotheses, highlight for the first time the interspecific range of the effect of larval cues.

Since larval cues have a cross-specific retentive effect, these ground-left odors could play the role of an interspecific aggregation vector. Indeed, the interspecific range of the effect could explain the ability of blowfly larvae of different species to socially aggregate together, as observed under field conditions and experimentally demonstrated by Boulay *et al*. (2016)^12^. Since the presence of a larval odor indicates the close presence of other larvae, the ability of a larva to preferentially stay in a marked area would increase the likelihood of interspecific aggregation. Such a mechanism has already been observed within two lepidopteran species, *Malacosoma disstria* and *M. americanum*^32^. Caterpillars of these species leave cues locally that affect not only their conspecifics but also the other species and lead to their gathering^32^.

When comparing sides marked by different species, larval preferences were different depending on the species and the cues concentration. At low concentrations (i.e. marking with five larvae), both *L. sericata* and *C. vomitoria* larvae showed no species-specific preference. But at high concentrations (i.e. marking with forty larvae), *L. sericata* larvae significantly preferred the side marked by conspecifics. The choice made by *L. sericata* larvae demonstrates that these larvae can discriminate cues depending on the emitting species. This ability seems to exist also in *C. vomitoria*, as the differences in both time spent and distance travelled between the two sides were very close to the significance level (respectively, P = 0.10 and P = 0.06). However, the fact that larval preferences were not observed at low concentration suggests the existence of a minimum perception threshold, below which larvae are not able to distinguish differences between cues. Such perception thresholds have already been evidenced in other Diptera larvae, for example in *Drosophila*^33^. Furthermore, the superior retentive effect of *L. sericata* larval cues shows that the strength of the effect can vary according to the emitting species and that different species can respond differently to conspecific cues.

The chemical compounds involved in the larval cues were likely present on the larval cuticle. As evidenced by control experiments, the effect of cues could not be induced by compounds or microorganisms coming from the environment and remaining on the larval integument (see Supplementary Fig. S2). The ability of larvae to discriminate cues between emitting species reinforces this observation. Consequently, a likely explanation of the source of larval cues is that these cues were produced by larvae and left during crawling (probably in a passive way). In many insect species, cuticular extracts (mostly hydrocarbons) initiate aggregation of individuals. This has been shown in ladybirds^34^ as well as in cockroaches^35^. Moreover, coexistence between different populations, colonies or species is often linked to similarities in cuticular compounds of individuals (e.g., refs^36–38^). Thus, as both *L. sericata* and *C. vomitoria* larvae can perceive cues from both species, these cues could contain some similar compounds. But as larvae can also discriminate cues when a minimum concentration is reached, some compounds may also quantitatively or qualitatively differ between the species. Two former studies analyzed the cuticular hydrocarbons at all developmental stages in *L. sericata*^39^ and *C. vomitoria*^40^. The hydrocarbons described were only linear alkanes. In *C. vomitoria* third instar larvae, the most abundant alkanes were C21, C22 and C25^40^, while in *L. sericata* they were C25, C27, C29 and C31^39^. Therefore, among the most abundant alkanes, only C25 were common to both species. The other alkanes ranging from C21 to C31 were almost all detected in both species but in very low proportions. According to these data, one hypothesis explaining both the interspecific perception and the concentration-dependent discrimination of cues is that larvae are able to detect linear alkanes from a vast range of size and to distinguish them depending on their size only if their concentration exceeds the value of the minimum perception threshold. Experiments using extracts or single compounds could allow to determine precisely which compounds are involved in larval aggregations.

Lastly, we observed that larvae spent more time in the side marked by a greater number of larvae (conspecific or heterospecific), confirming our third hypothesis. This behavior indicates a proportional effect between the attractive/retentive larval cues and the number of larvae that left it. Ultimately, it implies that larvae could detect *a posteriori* which place was the more crowded. This proportionality of cue effect to its intensity is in accordance with the self-organization theory. By increasing the probability of retaining individuals, the larval cues could allow self-amplification, resulting in a reinforcement of aggregation and a constant increase in the larval number. Such a density-dependent enhancement has been demonstrated in ants^2^, cockroaches^41^ and woodlice^1^. Furthermore, the interspecific effect of this mechanism would promote large interspecific aggregation. Broly *et al*. (2016)^42^ showed that woodlice of the species *Porcellio scaber* and *Oniscus asellus* were more likely to gather together when the group was composed of a greater number of individuals. In blowflies, large interspecific aggregates are clearly visible under field conditions and have been reported by many authors (e.g., refs^16,18,43^). Until now, the main explanatory factor for such a mixing of species was the clustering of eggs in places with a high nutritional value such as the face or wounds^7,44^. Together with the study of Boulay *et al*. (2016)^12^, our results add a new explanation to interspecific aggregations by providing a first experimental evidence of a mechanism producing a social aggregation of necrophagous larvae from different species.

Interspecific aggregation should provide benefits for each of the involved species, implying a low level of interspecific competition. Such benefits may be similar to those of intraspecific aggregation (e.g. more efficient feeding and development). Thus, collective behavior could allow the aggregated species to benefit from a rich and abundant but ephemeral and not easily digested food source^7^. Several authors have demonstrated that aggregation allows larvae to create a larval mass effect (local heat emission)^17,18^ that may speed up their rate of development and reduce the time spent in the cadaver^22–24^. By increasing their number, larvae may also improve food acquisition by extra-corporal digestion. Such an exodigestion process may be promoted by several factors involving numerous larvae such as elevated local temperatures, releasing of enzymes, changing of the local pH, control of bacterial activity and mechanically liquefying of flesh^7,45–47^. Such an interspecific Allee effect has never been formally demonstrated but is a likely reason for interspecific communication and aggregation of necrophagous calliphorid larvae. Larvae might also benefit from being aggregated due to the collective decisions made by the aggregated larvae, leading for example to find the best feeding sites^12,48^. Moreover, the stronger effect of *L. sericata* cues compared to *C. vomitoria* cues suggests that larvae may receive more benefits in aggregating with *L. sericata* than with *C. vomitoria*. Accordingly, *L. sericata* could provide an advantage for larvae that *C. vomitoria* would not have, such as effective digestive enzymes or effective antimicrobial secretions. Indeed, the antibacterial properties of *L. sericata* excretions/secretions (ES) have already been shown to differ from those of another blowfly species, *Calliphora vicina*, with a greater efficiency of *L. sericata* ES compared to *C. vicina* ES against some species of bacteria^49^. Another hypothesis is that *C. vomitoria* larvae have greater competitive abilities than *L. sericata*, allowing them to outcompete *L. sericata* in interspecific aggregates. For now, competition studies between these two species are lacking to confirm or refute this hypothesis.

In conclusion, this study is the first demonstration that *L. sericata* and *C. vomitoria* larvae leave on the ground a cue inducing an effect that is retentive, has an interspecific range, is proportional to its intensity and whose the strength varies depending on the emitting species. According to the self-organization theory, this effect could enhance the aggregation of larvae and promote interspecific aggregation. However, this mark is currently known only through its behavioral effect and has not been chemically identified. While cuticular hydrocarbons are likely candidates, this still lacks direct evidence. In addition, other vectors such as thigmotactism^7,26^, volatile odors^50^, substrate modification^46,47^ or thermal orientation^51^ could also be involved in interspecific larval aggregations in natural environment.

## Electronic supplementary material

Supplementary information
