# Supplementary material for: Communication in necrophagous Diptera larvae: interspecific effect of cues left behind by maggots and implications in their aggregation
Source: Sci Rep. 2018 Feb 12;8:2844. doi: 10.1038/s41598-018-21316-x (PMC5809460; doi:10.1038/s41598-018-21316-x)
Supplement: Supplementary file 1 — Supplementary information [file 41598_2018_21316_MOESM1_ESM.doc]

**Communication in necrophagous Diptera larvae: interspecific effect of cues left behind by maggots and implications in their aggregation**

**Quentin FOUCHE^*1^, Valery HEDOUIN^1^, and Damien CHARABIDZE^1^**

^1^CHU Lille, EA 7367 - UTML - Unite de Taphonomie Medico-Legale, University of Lille, 59000 Lille, France

^*^quentin.fouche@gmail.com

**SUPPLEMENTARY INFORMATION**

**Test of the experimental set-up**

Material and Methods

To test for the absence of bias in the setup, five homogeneous combinations were tested: control (i.e., no marking, "control vs. control"), 5 *L. sericata* ("5 *L. sericata* vs. 5 *L. sericata*"), 5 *C. vomitoria* ("5 *C. vomitoria* vs. 5 *C. vomitoria*"), 40 *L. sericata* ("40 *L. sericata* vs. 40 *L. sericata*") and 40 *C. vomitoria* ("40 *C. vomitoria* vs. 40 *C. vomitoria*").

Results

For these homogeneous conditions and for both species, no significant differences in the larval displacement between the two sides of the arena were observed (time spent in the side, distance, and average speed) (Supplementary Table S1, Supplementary Fig. S1).


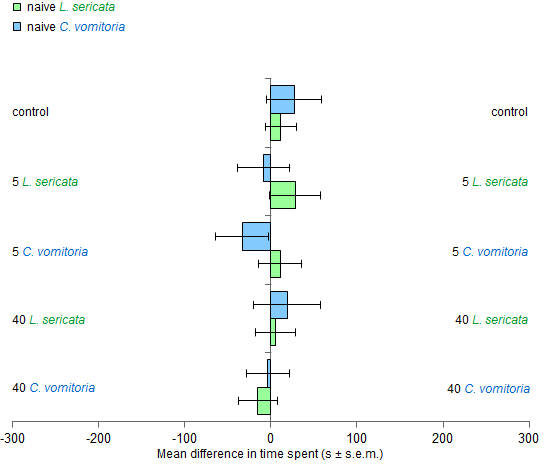


**Supplementary Figure S1.** Mean differences (right side minus left side, mean ± s.e.m.) in time spent between the two sides of the arena in homogeneous marking condition. The results obtained with naive *L. sericata* larvae are reported in green, while those for *C. vomitoria* are in blue. 30 replicates were performed for each condition.

Discussion

The absence of significant preference for either side of homogenously marked arenas demonstrates that the experimental device did not include a spatial bias: the positions of the two sides did not affect larval choice. Therefore, preferences observed in heterogenous marked arenas can be explained exclusively by the differences in marking between the two sides.

**Test of the cleaning method**

Several studies have shown that Dipteran larvae can perceive food odors and to move toward their source^1,2,3^. This capacity may introduce a bias in binary choice tests: if some food traces are left by marking larvae, tested larvae may be attracted to the marked side regardless of the presence of a species-specific cue. To eliminate this potential bias, marking larvae were cleaned before each test by confining them in a pillbox containing moistened pine sawdust for 4 hours.

Material and Methods

To check the efficiency of this cleaning method, two tests were performed with marking larvae that were intensively washed. This intensive washing treatment was used to remove all food remains that may have been present on the larval cuticle. For this purpose, larvae were first confined in a pillbox containing moistened pine sawdust for 24 hours, then placed in a tube with 1.5 ml of distilled water and shaken three times for one second using a vortex (IKA MS2 Minishaker). The larvae were finally confined again for 2 hours in a pillbox with moistened filter paper before being used for the experiments. The two tested conditions were "five 24 h-cleaned *L. sericata* vs. five 4 h-cleaned *L. sericata*" (tested with *C. vomitoria* larvae) and "5 of 24 h-cleaned *C. vomitoria* vs. 5 of 4 h-cleaned *C. vomitoria*" (tested with *L. sericata* larvae).

Results

The time spent, the distance traveled and the average speed of *C. vomitoria* larvae were not significantly different between the two sides. The same results were obtained with naïve *L. sericata* larvae using a *C. vomitoria* mark (Supplementary Table S1, Supplementary Fig. S2).


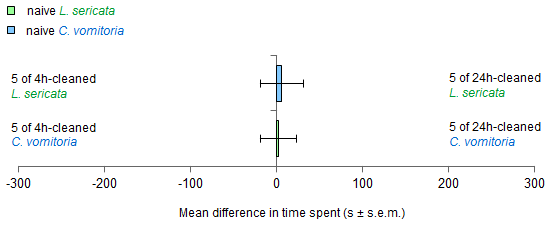


**Supplementary Figure S2.** Mean differences (mean ± s.e.m.) in time spent between the two sides of the arena in heterogeneous marking condition (4 h-cleaned vs 24 h-cleaned marking larvae). The time difference was calculated by subtracting the time spent on the side marked by 4 h confinement larvae (left) from the time spent on the side marked by intensively washed larvae (right). The results obtained with naive *L. sericata* larvae are reported in green, while those for naive *C. vomitoria* are in blue. 30 replicates were performed for each condition.

| Cues tested | side 1 | control | 5 *L. sericata* | 5 *C. vomitoria* | 40 *L. sericata* | 40 *C. vomitoria* | 4 h-cleaned  *L. sericata* | 4 h-cleaned  *C. vomitoria* |
| --- | --- | --- | --- | --- | --- | --- | --- | --- |
|  | side 2 | control | 5 *L. sericata* | 5 *C. vomitoria* | 40 *L. sericata* | 40 *C. vomitoria* | 24 h-cleaned  *L. sericata* | 24 h-cleaned  *C. vomitoria* |
| Time spent  (s) | *L. sericata* | 156 ± 9  144 ± 9  t = 0.63; NS | 164 ± 15  136 ± 15  t = 0.95; NS | 155 ± 13  145 ± 13  t = 0.42; NS | 153 ± 12  147 ± 12  t = 0.22; NS | 142 ± 11  158 ± 11  t = -0.70; NS | - | 151 ± 11  149 ± 11  t = 0.08; NS |
|  | *C. vomitoria* | 164 ± 16  136 ± 16  t = 0.86; NS | 146 ± 15  154 ± 15  t = -0.28; NS | 133 ± 15  167 ± 15  t = -1.10; NS | 159 ± 19  141 ± 19  V = 255; NS | 148 ± 12  152 ± 12  t = -0.14; NS | 147 ± 12  152 ± 12  t = -0.23; NS | - |
| Distance travelled  (cm) | *L. sericata* | 48 ± 3  44 ± 3  t = 0.85; NS | 44 ± 4  38 ± 4  t = 0.92; NS | 45 ± 3  42 ± 4  t = 0.45; NS | 41 ± 3  38 ± 3  t = 0.36; NS | 38 ± 3  41 ± 3  t = 0.50; NS | - | 48 ± 3  45 ± 3  t = 0.38; NS |
|  | *C. vomitoria* | 58 ± 6  45 ± 5  t = 1.27; NS | 44 ± 5  47 ± 5  t = -0.32; NS | 42 ± 5  51 ± 4  t = -1.09; NS | 46 ± 5  43 ± 6  V = 252; NS | 53 ± 5  54 ± 4  t = 0.03; NS | 42 ± 4  42 ± 4  t = -0.13; NS | - |
| Average speed  (cm/s) | *L. sericata* | 0.31 ± 0.01  0.31 ± 0.01  t = 0.33; NS | 0.27 ± 0.01  0.28 ± 0.01  t = -0.16; NS | 0.31 ± 0.01  0.29 ± 0.01  V = 298; NS | 0.27 ± 0.01  0.26 ± 0.01  t = 1.03; NS | 0.27 ± 0.01  0.27 ± 0.01  t = 0.07; NS | - | 0.32 ± 0.01  0.31 ± 0.01  t = 0.57; NS |
|  | *C. vomitoria* | 0.36 ± 0.01  0.33 ± 0.02  t = 1.90; NS | 0.32 ± 0.02  0.30 ± 0.02  t = 1.08; NS | 0.33 ± 0.03  0.32 ± 0.01  V = 177; NS | 0.31 ± 0.01  0.32 ± 0.02  V = 144; NS | 0.37 ± 0.01  0.37 ± 0.01  t = 0.29; NS | 0.28 ± 0.01  0.28 ± 0.01  t = -1.71; NS | - |

**Supplementary Table S1.** Mean values of larval displacement in the two sides of the arena for homogeneous marking conditions (same cues on both sides of the arena). For each condition, "side 1" is reported first, "side 2" is underneath, and statistical values are reported on the last line (NS: not significant). Dashes indicate that no experiments were conducted for the condition. 30 replicates were performed for each condition.

Discussion

The absence of preference between a side marked by 24 h-cleaned larvae and a side marked by 4 h-cleaned larvae confirms the hypothesis that a 4 h confinement in moist pine wood dust was sufficient to successfully remove any potential food traces from the cuticle of larvae. If the larval cues were only composed of food remains, larvae would have spent significantly more time in the side marked by the 4 h-cleaned larvae.

Other arguments contribute to invalidating the hypothesis of food bias. First, the existence of hydrocarbons on the larval cuticle has already been shown (e.g., refs 4,5). While it has not yet been shown that these hydrocarbons act as aggregation vectors, this hypothesis is likely; Boulay et al. (2013)^1^ demonstrated the existence of an aggregation vector allowing larvae of the same species to make collective decisions. Furthermore, these authors also performed binary-choice experiments in an arena fully soaked with food extract. Even under such conditions, larvae still showed a preference for the side of the arena previously marked by larvae, thus invalidating the hypothesis of a cuticular food odor^1^. Together, these results strongly suggest the existence of a cuticular cue different from food traces, left on the ground by larvae and affecting the behavior of other necrophagous larvae.

**Additional analyses**

Material and Methods

Two additional parameters were calculated in each side of the heterogeneous conditions: (1) the number of experiments in which the larva started to move in the respective side and (2) the curvature of the larval path. To determine in which side the larva began to move first, the distance from the larva to the limit between sides was measured. By definition the larva was only considered to start to move in a side when this distance was at least 3 mm within this side. For the path curvature, the mean meander of the path was calculated by dividing the mean absolute rotation angle of the path in a side by the total distance travelled in this side. As the values were excessively high when the distance travelled was low, only the values for which the distance travelled was greater than 2 cm were kept for the analyses. Comparisons of the number of larvae between sides were performed using the Pearson's Chi-squared test and comparisons of the mean meander between sides were performed using the Student's t test when normality and homoscedasticity were present (respectively evaluated by the Shapiro’s test and the Fisher's exact test) or using the Mann-Whitney U test when these conditions were not fulfilled.

Results

For each heterogeneous condition, the number of experiments where larvae started to move in one side and the path curvature were not significantly different between the two sides (Supplementary Table S2, Supplementary Fig. S3 and S4).


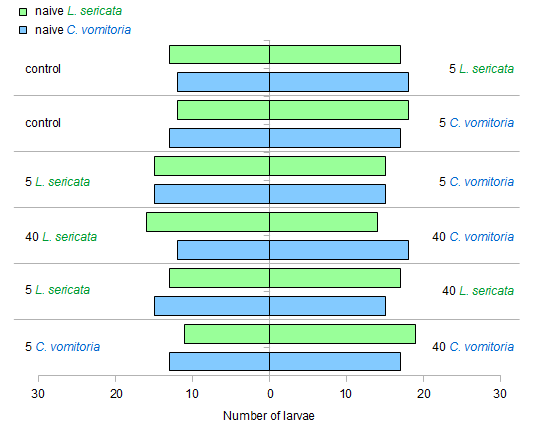
**Supplementary Figure S3.** Number of experiments where *L. sericata* (green) and *C. vomitoria* (blue) larvae started to move in each side of the heterogeneous conditions. 30 replicates were performed for each condition.


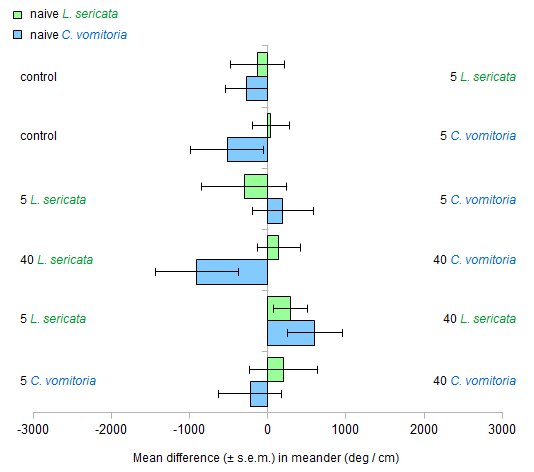


**Supplementary Figure S4.** Mean difference (mean ± s.e.m.) in meander (deg / cm) of *L. sericata* (green) and *C. vomitoria* (blue) larvae between the two sides of the arena in heterogeneous conditions. The difference was calculated by subtracting the meander on the left side of the graph from the meander on the right side. 30 replicates were performed for each condition.

| Cues tested | side 1 | control | control | 5 *L. sericata* | 40 *L. sericata* | 5 *L. sericata* | 5 *C. vomitoria* |
| --- | --- | --- | --- | --- | --- | --- | --- |
|  | side 2 | 5 *L. sericata* | 5 *C. vomitoria* | 5 *C. vomitoria* | 40 *C. vomitoria* | 40 *L. sericata* | 40 *C. vomitoria* |
| Number of experiments in which the larva started to move in a side | *L. sericata* | 13  17  X = 0.53; NS | 12  18  X = 1.20; NS | 15  15  X = 0; NS | 16  14  X = 0.13; NS | 13  17  X = 0.53; NS | 11  19  X = 2.13; NS |
|  | *C. vomitoria* | 12  18  X = 1.20; NS | 13  17  X = 0.53; NS | 15  15  X = 0; NS | 12  18  X = 1.20; NS | 15  15  X = 0; NS | 13  17  X = 0.53; NS |
| Meander (deg / cm) | *L. sericata* | 1665 ± 382  1674 ± 271  W = 351; NS | 1212 ± 213  1511 ± 281  W = 480; NS | 2705 ± 386  2334 ± 412  W = 317; NS | 919 ± 131  982 ± 292  W = 349; NS | 1616 ± 239  1892 ± 279  W = 484; NS | 1457 ± 312  1726 ± 346  W = 426; NS |
|  | *C. vomitoria* | 2177 ± 447  2156 ± 420  W = 298; NS | 2648 ± 428  2762 ± 517  W = 297; NS | 2626 ± 308  2821 ± 433  W = 324; NS | 1987 ± 391  1141 ± 193  W = 232; NS | 2864 ± 512  3203 ± 461  W = 225; NS | 3033 ± 424  2834 ± 318  t = -0.38; NS |

**Supplementary Table S2.** Values of the number of experiments in which the larva started to move in a side and mean values of the larval path meander in the two sides of the arena for heterogeneous conditions. For each condition, "side 1" is reported first, "side 2" is underneath, and statistical values are reported on the last line (NS: not significant). 30 replicates were performed for each condition.

**References**

1. Boulay, J., Devigne, C., Gosset, D. & Charabidze, D. Evidence of active aggregation behaviour in *Lucilia sericata* larvae and possible implication of a conspecific mark. *Anim. Behav.* **85**, 1191–1197 (2013).
2. Cobb, M. What and how do maggots smell?. *Biol. Rev.* **74**, 425-459 (1999).
3. Christopherson, C. & Gibo, D. L. Foraging by food deprived larvae of *Neobellieria bullata* (Diptera: Sarcophagidae). *J. Forensic Sci.* **42**, 71-73 (1997).
4. Gołębiowski, M. *et al*. Cuticular and internal n-alkane composition of *Lucilia sericata* larvae, pupae, male and female imagines: application of HPLC-LLSD and GC/MS-SIM. ***Bull. Entomol. Res.*** **102**, 453–460 (2012).
5. Roux, O., Gers, C. & Legal, L. Ontogenetic study of three Calliphoridae of forensic importance through cuticular hydrocarbon analysis. *Med. Vet. Entomol.* **22**, 309–317 (2008).
